# The X-Ray Emission Effectiveness of Plasma Mirrors: Reexamining Power-Law Scaling for Relativistic High-Order Harmonic Generation

**DOI:** 10.1038/s41598-020-61255-0

**Published:** 2020-03-20

**Authors:** Matthew R. Edwards, Julia M. Mikhailova

**Affiliations:** Princeton University, Department of Mechanical and Aerospace Engineering, Princeton, New Jersey 08544 USA

**Keywords:** Ultrafast lasers, High-harmonic generation, X-rays, Laser-produced plasmas

## Abstract

Ultrashort pulsed lasers provide uniquely detailed access to the ultrafast dynamics of physical, chemical, and biological systems, but only a handful of wavelengths are directly produced by solid-state lasers, necessitating efficient high-power frequency conversion. Relativistic plasma mirrors generate broadband power-law spectra, that may span the gap between petawatt-class infrared laser facilities and x-ray free-electron lasers; despite substantial theoretical work the ultimate efficiency of this relativistic high-order-harmonic generation remains unclear. We show that the coherent radiation emitted by plasma mirrors follows a power-law distribution of energy over frequency with an exponent that, even in the ultrarelativistic limit, strongly depends on the ratio of laser intensity to plasma density and exceeds the frequently quoted value of  −8/3 over a wide range of parameters. The coherent synchrotron emission model, when adequately corrected for the finite width of emitting electron bunches, is not just valid for p-polarized light and thin foil targets, but generally describes relativistic harmonic generation, including at normal incidence and with finite-gradient plasmas. Our numerical results support the *ω*^−4/3^ scaling of the synchrotron emission model as a limiting efficiency of the process under most conditions. The highest frequencies that can be generated with this scaling are usually restricted by the width of the emitting electron bunch rather than the Lorentz factor of the fastest electrons. The theoretical scaling relations developed here suggest, for example, that with a 20-PW 800-nm driving laser, 1 TW/harmonic can be produced for 1-keV photons.

## Introduction

Plasma mirrors – microscopically small, transient reflectors created by pulsed laser beams irradiating and ionizing solid surfaces – are powerful photonic devices for manipulating light. When driven by laser pulses with relativistic intensities (${I}_{{\rm{rel}}}=1.37\times 1{0}^{18}{\lambda }_{[{\rm{\mu }}{\rm{m}}]}^{-2}$ W/cm^2^ for wavelength *λ*) and high temporal contrast, plasma mirrors can emit short-wavelength coherent light with broad bandwidth, attosecond duration, and high pulse energy, rivaling the brightness of existing large-scale sources of coherent extreme ultraviolet and x-ray radiation^1,2^, and showing promise for capturing electronic dynamics on the attosecond timescale^3,4^. This emission, relativistic high-order harmonic generation (RHHG), has been extensively studied with analytic and computational approaches^5–17^ and observed in a number of successful experiments^18–31^. Here we use a large set of calculated interactions to relate the RHHG spectrum – specifically the power-law relations in the distribution of energy over frequency and the cutoff that marks the onset of steeper decay – to the dynamics of the relativistic electrons accelerated by the laser and plasma fields.

The first high-order harmonics were generated from solid-density plasmas in the sub-relativistic regime^32–38^, where the highest frequencies produced are limited by the plasma frequency^39–41^. For relativistic intensities the nonlinear frequency conversion mechanism is fundamentally different, allowing much higher efficiencies and generating harmonics well above the plasma frequency^5,6^; experimentally demonstrated photon energies have reached 3.8 keV ( ≈3200 harmonics)^23^. Advances in laser technology are now making attosecond sources based on RHHG feasible^42^. The relativistic harmonic generation mechanism is often described as a relativistically oscillating mirror (ROM), where the incident laser field is Doppler upshifted by the longitudinal motion of the reflective plasma surface^6,10^, though for strong laser-plasma interactions sub-cycle energy storage in the plasma becomes important and a more detailed treatment of the electron motion is required, e.g. the relativistic electron spring (RES)^12,43,44^ or coherent synchrotron emission (CSE)^45–47^ models. In the CSE model, dense nanometer-scale bunches of electrons accelerated by the laser and plasma fields in relativistic trajectories radiate high frequency light; perpendicular acceleration and velocity near the time of emission produce a synchrotron-like spectrum. An emitting electron bunch is created once per laser cycle – or once per half cycle for normal incidence – and the radiation from each cycle arrives as an attosecond burst of broadband light. Determining the conversion efficiency of relativistic RHHG is particular goal of both theoretical and computational work, because it directly governs the viability of RHHG as an ultraviolet and x-ray source.

**Figure 1:**
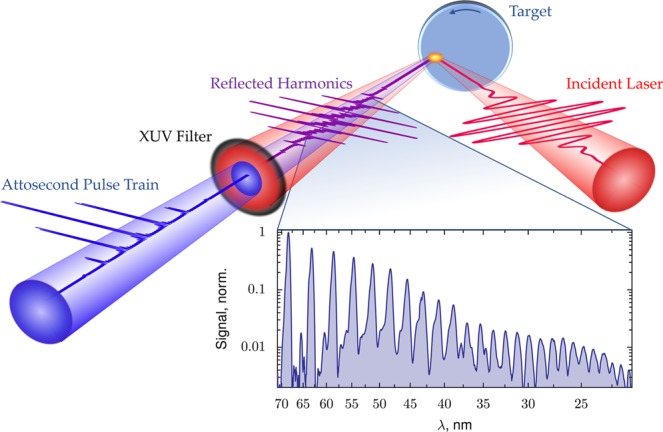
Schematic of relativistic harmonic generation with a p-polarized ultrafast laser incident on a solid target. The spectrum was experimentally measured using the Princeton 20 TW Ti:sapphire laser system (Amplitude Technologies), with 70 mJ incident on a BK7 target in 25 fs to produce a peak intensity of 8 × 10^19^ W/cm^2^. The central fundamental wavelength is 800 nm. The reflected single-shot spectrum was passed through a 150 nm aluminum filter to remove the fundamental and imaged with a flat-field diffraction-grating spectrometer. The plasma frequency limits sub-relativistic harmonics to 40 nm. Note that the harmonic structure appears because the driving laser pulse is multi-cycle; a single-cycle driver would produce an isolated attosecond pulse and a continuum spectrum.

**Figure 2:**
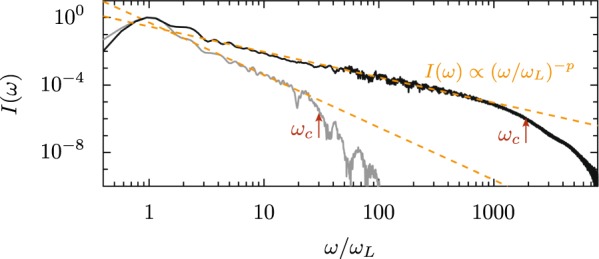
Calculated RHHG spectra (PIC). Upper spectrum: *a*_0_ = 1000, *N* = 4000, *p* = 1.5, and *ω*_*c*_/*ω*_*L*_ = 1900. Lower spectrum: *a*_0_ = 20, *N* = 300, *p* = 3.1, and *ω*_*c*_/*ω*_*L*_ = 30. For both, *θ*_*L*_ = 45°, *τ* = 3 fs.

Relativistic high-order harmonics are generated in the direction of specular reflection when a relativistic-intensity laser is focused on a solid density target; a schematic of the process is shown in Fig. 1 together with an experimentally measured spectrum. The driving laser, focused to intensities greater than 10^18^ W/cm^2^, produces a non-linear response from the solid-density target, creating harmonics of the initial frequency. Simulations suggest that these harmonics are phase-locked, leaving an attosecond pulse train if the low-order frequencies are filtered out of the beam. Example spectra for single-cycle driving pulses calculated via particle-in-cell (PIC) simulations are shown in Fig. 2. The spectral intensity of the reflected spectrum *I*(*ω*) decreases as frequency increases. PIC simulations and several analytic models predict that this decrease follows a power law [*I*(*ω*) ∝ (*ω*)^−*p*^] up to a cutoff frequency (*ω*_*c*_), beyond which the intensity falls much more rapidly. Under some conditions more complex behavior appears, including spectral modulations^19,48^. Since the efficiency – and by extension the usefulness – of RHHG rests on how rapidly the spectrum decreases, a great deal of analytic effort has been devoted to predicting the slope of the power-law decay and the cutoff frequency. Early computational work by Gibbon^5^ found harmonics up to the water window (2.3–4.4 nm) with an intensity-dependent exponent (*p*) between 6 and 3.5. This was followed by Gordienko *et al*.^8^, who used the oscillating mirror model to posit universal values of *p* = 2.5 and *p* = 3 for quasi-monochromatic and broadband pulses, respectively, with a cutoff proportional to *γ*^2^, where *γ* is the Lorentz factor of the surface electrons. Baeva *et al*. adjusted the oscillating mirror model to account for spikes in the gamma factor at the reflecting surface, leading to *p* = 8/3 power scaling up to a cutoff proportional to *γ*^3^^10^. With predictions of *p* = 4/3 or 6/5 from the CSE model^45^, suggestions from the RES model that the spectrum is exponential^12^, and debate about the validity of any universal law^49^, proposed values of *p* span a broad range, with strong implications for the usefulness of RHHG-based x-ray sources.

**Figure 3:**
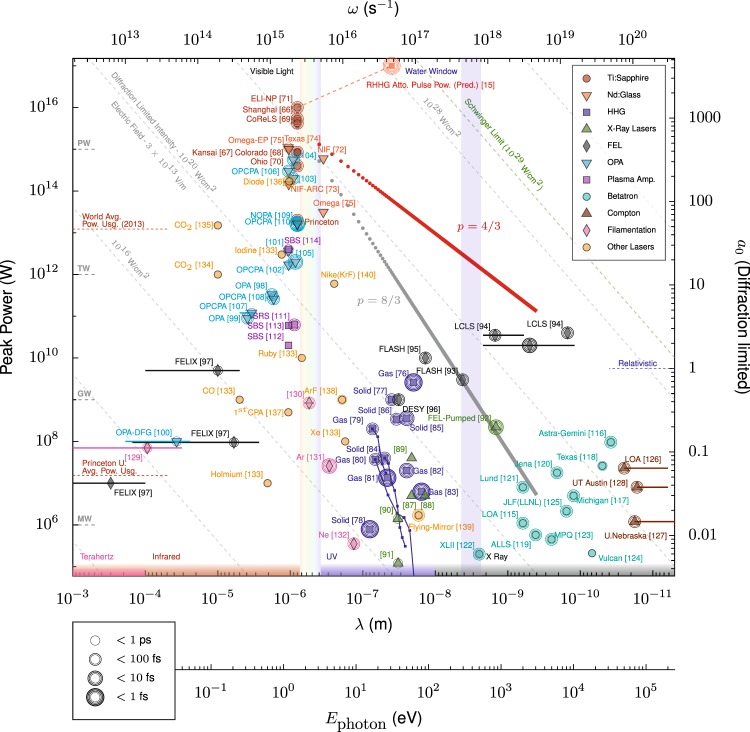
Experimentally-demonstrated coherent radiation sources with high peak power compared to relativistic harmonic scaling laws. Pulse duration is indicated by the number of circles around each point. Diagonal lines mark the diffraction limited intensity (*f*/1 focusing) for light of particular maximum power and wavelength. The RHHG scaling is based on peak power in each integer harmonic interval; for individual harmonics the pulse duration will match the driving laser. Points are taken from results in refs. ^66–140^. The key distinguishes sources derived from Ti:sapphire or Nd:glass laser architectures, RHHG (high-order-harmonic generation), FEL (free electron laser), OPA (optical parametric amplification) and OPCPA (optical parametric chirped pulse amplification), plasma amplification [including stimulated Raman (SRS) and Brillouin (SBS) scattering], betatron radiation and Compton scattering from laser-accelerated electrons – for review, see^141^ – and laser-filamentation based frequency conversion. Solid lines indicate a nominal tuning range. Note that for RHHG sources if an attosecond pulse duration was not measured, the power was estimated from the incident pulse duration and harmonic energy. The RHHG scalings assume a 10-PW driver and neglect absorption.

The limiting values of the power-law exponent and spectral cutoff are critically important for determining how effectively near-infrared light can be converted to extreme ultraviolet (XUV) and x-ray radiation by RHHG. Contemporary sources of high-peak-power radiation include solid-state lasers providing petawatt power with near-infrared light, free electron lasers producing high-flux x-rays, and high-order-harmonic generation, supporting attosecond-duration pulses. Figure 3 plots a selection of experimentally achieved peak powers against wavelength, encompassing a broad range of coherent light sources. Relativistic harmonics are normally driven by near-infrared or visible light, where the highest powers and intensities are available from solid state Ti:sapphire, OPCPA, and Nd:glass lasers, as shown by the cluster of points exceeding a petawatt in Fig. 3.

The importance of the power-law exponent value for the usefulness of RHHG is illustrated by the two theoretical scaling lines drawn on Fig. 3: *p* = 4/3 in red and *p* = 8/3 in grey. In both cases the normalization is found by assuming a 10-PW driver and neglecting absorption; the integral under the spectrum equals the input 10-PW power. For light produced at 0.8 nm by RHHG driven at 800 nm, the difference between *ω*^−4/3^ and *ω*^−8/3^ scaling is four orders of magnitude in power – for a petawatt-class driver, this is the difference between an x-ray RHHG source brighter than a free electron laser and one three orders of magnitude weaker. The scaling lines in Fig. 3 show the power in particular harmonics, but the reflected beam contains all harmonics simultaneously. Since the compression of energy into an attosecond pulse can increase the reflected intensity, the peak power achievable for the full spectrum of reflected radiation can be *higher* than that of the driving laser, making RHHG a possible route to intensities beyond the limits of CPA^12,50,51^. The power of an attosecond pulse which could be achieved from a 10 PW driver based on simulation results is also marked^15^.

We show that the power-law exponent *p* of the reflected spectrum is a continuous function of the experimental parameters, even in the relativistic limit, and that *p* can be less than 8/3 across a wide range of conditions. Drawing on particle-in-cell simulations, we delineate scaling relationships for the power-law exponent and cutoff and relate them to observed properties of surface electrons, including the Lorentz factor, in an effort to understand both the range of validity of proposed models and the reachable efficiencies of high-order-harmonic generation.

## The Power-Law Spectrum of RHHG

RHHG is primarily characterized by the spectrum of the reflected light. Most theoretical models and simulation results are consistent with a power-law spectral shape, although there is some disagreement regarding the appropriate exponent^5,8,10,45,49^. A power-law fit seems to reasonably approximate the computed spectra we observe. For each spectrum in Fig. 2, the initial power-law [$I(\omega )\propto {(\omega /{\omega }_{L})}^{-p}$, where *ω*_*L*_ is the laser frequency] eventually gives way to a steeper falloff. We will define the cuffoff frequency *ω*_*c*_ as the frequency at which the spectral intensity has dropped by a factor of *e* below that predicted by continuing the power-law scaling, a definition which is reasonably robust to the variation in spectral shape that can occur near the cutoff; *ω*_*c*_ is marked for the two spectra in Fig. 2. The value of *p* is important because it can be used to calculate an approximate value for the energy in each harmonic with *ω* < *ω*_*c*_. In this section, we consider how *p* varies due to the laser intensity and plasma density and relate this variation to the microscopic motion of plasma electrons.

Without atomic effects, the interaction physics may be scaled by the laser frequency, removing explicit dependence on *ω*_*L*_. The strength of the incident laser is described by its normalized field amplitude: *a*_0_ = *eE*/*m*_*e*_*ω*_*L*_*c*, where *E* is the maximum value of the electric field envelope, *m*_*e*_ and *e* are the mass and charge of an electron, and *c* is the speed of light. The plasma density (*n*_*e*_) is normalized by the critical density (${n}_{c}={m}_{e}{\omega }_{L}^{2}$/4*πe*^2^) to give *N* = *n*_*e*_/*n*_*c*_. Since the two forces of interest in the problem come from the laser and the plasma, the dynamics of relativistic RHHG are governed by the balance between the laser driving force (∝*a*_0_) and the plasma restoring force (∝*N*)^15^. The similarity parameter (*S* = *N*/*a*_0_)^52–54^ arises from nondimensionalization of the Vlasov-Maxwell equations in the relativistic limit (*a*_0_ ≫ 1) and generally plays an important role in relativistic laser-plasma interactions, although its exact relevance for RHHG has been questioned^49,55–57^. The generation of high frequency light via relativistic RHHG is a subcycle process, so to isolate the detailed mechanism we will primarily show results for single-cycle (Gaussian) driving pulses.

**Figure 4:**
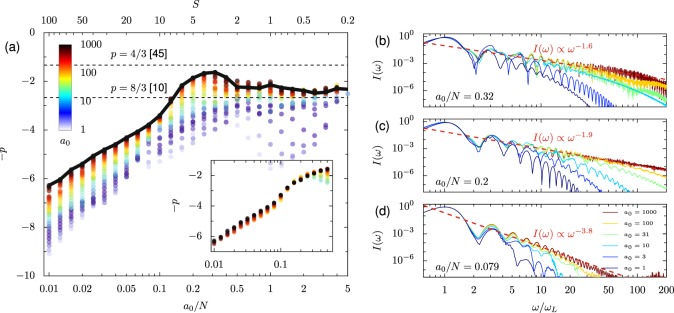
(**a**) Spectral scaling of the power-law exponent *p* where $I(\omega )\propto {(\omega /{\omega }_{L})}^{-p}$, for high-order harmonics generated at normal incidence with varied *a*_0_ and *N*. The exponent is found via a fit to the harmonic range 1 < *ω*/*ω*_*L*_ < 100. Inset: Only those points which satisfy *a*_0_/$\sqrt{N} > 2$. (**b–d**) Spectra recorded for selected *a*_0_ at (**b**) *a*_0_/*N* = 0.32, (**c**) *a*_0_/*N* = 0.2, and (**d**) *a*_0_/*N* = 0.079. The dashed red lines are power-law fits to the *a*_0_ = 1000 spectra.

Figure 4a shows the variation in *p* for 1 ≤ *a*_0_ ≤ 1000 and 0.01 ≤ *a*_0_/*N* ≤ 5 at angle of incidence *θ*_*L*_ = 0. The value of *p* is found from a linear fit to the logarithm of the spectrum vs the logarithm of the normalized frequency in the range 1 < *ω*/*ω*_*L*_ < 100; the fitting procedure is described in more detail in the Methods section. There are two key points of interest in Fig. 4a. First, for *a*_0_ → *∞* at fixed *a*_0_/*N*, *p* approaches a constant value that can be approximated as proportional to the logarithm of *a*_0_/*N*, i.e. ${{\rm{lim}}}_{a\to \infty }p({a}_{0}/N)\propto {\rm{ln}}\,({a}_{0}/N)$, for a wide range of values. Second, for *a*_0_/*N* > 0.15, *p* exceeds the predicted maximum scaling at normal incidence (*p* = 8/3)^10^. This corresponds to where the maximum reflected intensity exceeds the incident intensity. Note in particular that as *a*_0_/*N* is varied, a very large range of values for *p* can be reached even in the limit *a*_0_ → *∞*, from *p* larger than 6 to less than 2. The example spectra at right show how the spectrum of reflected radiation varies at fixed *a*_0_/*N* and varied *a*_0_ for selected values of *a*_0_/*N*. As *a*_0_ increases, the spectra follow the power law over a larger frequency range; the cutoff increases with *a*_0_.

At moderate values of *a*_0_ the fitted value of *p* can be far from the asymptotic limit. Neglecting deformation of the plasma surface, the electric field at the vacuum-plasma interface for normal incidence and *a*_0_ ≪ *N* can be approximated as: *E*_*S*,0_ = 2*E*_*I*,0_/$(1+\sqrt{1-N})$, so that *a*_0,eff_ ≈ 2*a*_0_/$\sqrt{N}$ for *N* ≫ 1. To derive this, note that at the plasma-vacuum boundary, the complex valued incident *E*_*I*,0_, reflected *E*_*R*,0_ and transmitted (*E*_*T*,0_) electric fields must satisfy *E*_*I*,0_ + *E*_*R*,0_ = *E*_*T*,0_ and the magnetic fields satisfy *B*_*I*,0_ + *B*_*R*,0_ = *B*_*T*,0_. In vacuum, *E*_0_ = *B*_0_, and the plasma dispersion relation gives ${E}_{T,0}=\sqrt{1-N}{B}_{T,0}$. The surface field *E*_*S*,0_ is simply *E*_*T*,0_ at the interface. Since the transverse Lorentz factor of electrons at the surface will be proportional to the surface field, the condition for relativistic motion is *a*_0_/$\sqrt{N}\gg 1$, not simply *a*_0_ ≫ 1. When we filter the plotted points by this condition, and fit *p* in the range 0 < *ω* < 20 to avoid the spectral cutoffs, we find that *p* is a function of only *a*_0_/*N* (Fig. 4a inset); there is no variation with *a*_0_ for lower-order harmonics. This suggests the relevance of the similarity model to RHHG and means we can attribute the deviations in *p* observed for smaller *a*_0_, particularly at smaller values of *a*_0_/*N*, to fact that the motion is not fully relativistic.

**Figure 5:**
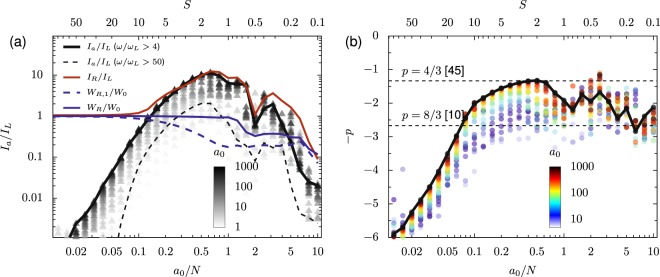
Attosecond pulse generation efficiency (**a**) and power-law spectrum scaling coefficient (**b**) for varied *a*_0_/*N* at *θ*_*L*_ = 30°. In (**a**), *I*_*a*_ for the points is calculated using a window 4 < *ω*∕*ω*_*L*_ < 100. The solid black line shows *I*_*a*_/*I*_*L*_ in the large *a*_0_ limit, i.e. *a*_0_ = 1000. The solid red line shows the total reflected intensity, and the dashed black line shows the attosecond pulse efficiency for 50 < *ω*/*ω*_*L*_ in the same limit. The solid blue line shows the reflected energy and the dashed blue line shows the reflected energy in the fundamental, (*ω*/*ω*_*L*_ < 1.5), both for *a*_0_ = 1000. In (**b**), the solid black line marks *p* for *a*_0_ = 1000, and the dashed lines correspond to *p* = 4/3^45^ and *p* = 8/3^10^. The incident pulses are single cycle.

The same basic dependence of the spectral power law on *a*_0_ and *N* persists at non-zero angle of incidence for P-polarized light, shown, for example, at *θ*_*L*_ = 30° in Fig. 5b. As for *θ*_*L*_ = 0°, *p* approaches a constant value for *a*_0_ → *∞* at fixed *a*_0_/*N* and decreases for *a*_0_/*N* < 0.5. Although for both *θ*_*L*_ = 0° and *θ*_*L*_ = 30° the fitted value of *p* does not quickly increase for *a*_0_/*N* > 1, Fig. 5a shows that the attosecond pulse generation efficiency (*I*_*a*_/*I*_*L*_) reaches a maximum near *a*_0_/*N* ≈ 0.5 and falls for larger *a*_0_/*N*. This decrease occurs for all spectral filters, e.g. 0 < *ω*/*ω*_*L*_ (red), 4 < *ω*/*ω*_*L*_ < 100 (black, solid), 50 < *ω*/*ω*_*L*_ (black, dashed), as well as for the total reflected energy (solid blue) and reflected fundamental energy (dashed blue). This corresponds to the relativistic transparency regime, where *a*_0_ ≫ *N* and the high-intensity laser pulse will propagate into plasma which is overdense for less-intense light. In this regime, the laser force overpowers the plasma restoring forces, leading to inefficient RHHG. The relatively shallow values of *p* for *a*_0_/*N* > 1 do not capture the total decrease in the spectrum as a result of the transparency of the plasma in this regime. The most efficient attosecond pulse generation is achieved when the laser and plasma forces are close to balanced.

**Figure 6:**
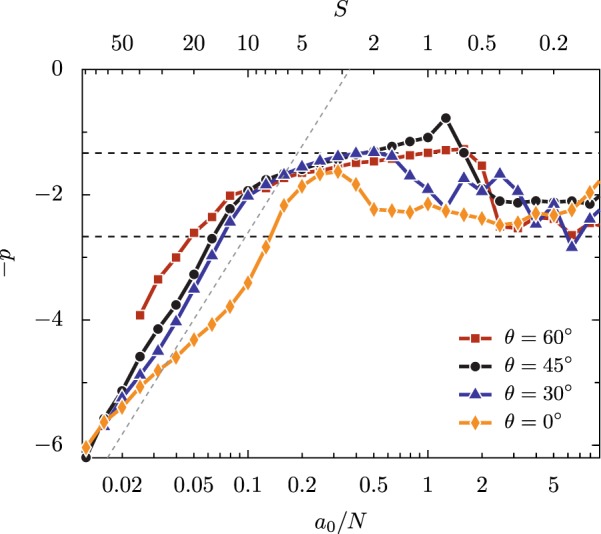
Variation in the spectral power-law exponent *p* with *a*_0_/*N* for varied angle at *a*_0_ = 1000, an approximation for the limit *a*_0_ → *∞*. All points were calculated with separate PIC simulations using single-cycle driving pulses. The horizontal lines mark *p* = 8/3 and 4/3. The diagonal line shows $p=2{\rm{ln}}\,(N/{a}_{0})+C$.

As the angle of incidence (*θ*_*L*_) changes, the dependence of *p* on *a*_0_/*N* as *a*_0_ → *∞* remains relatively consistent. Some variation in the exact values of *p* that are found at a given *a*_0_/*N* can be seen in Fig. 6, where the *a*_0_ → *∞* limit is approximated by *a*_0_ = 1000. For *a*_0_/*N* < 0.5, *p* can be very roughly estimated, to within  ±1, by $p\approx 2{\rm{ln}}\,(N/{a}_{0})+C$, shown by the diagonal dashed line. More importantly, at all angles considered (*θ*_*L*_ = 0°, 30°, 45°, and 60°) the shallowest values of *p* lie in the interval 0.1 < *a*_0_/*N* < 1, with a limit near *p* = 4/3. Apart from a few points for *θ*_*L*_ = 45°, this  −4/3 line appears to be a limit for the power-law exponent across all of the angle of incidence, intensities, and densities considered here.

## Electron Trajectories

The  −4/3 spectral scaling arises from the CSE model of high-order harmonic generation^45^ by assuming that the radiation of interest is emitted by a single bunch of electrons over a time when its position in the direction of specular reflection can be approximated for small *t* as:

1 $$x(t)={\beta }_{x}t{\omega }_{L}+\frac{{\alpha }_{1}}{3}{(t{\omega }_{L})}^{3}$$

and its transverse current varies linearly as:

2 $${j}_{y}(t)={\alpha }_{0}t{\omega }_{L}$$

where *t* = 0 is the time of emission, *β*_*x*_ = *v*_*x*_/*c* is the normalized velocity component in the *x* direction, and *α*_0_ and *α*_1_ are constants. Using the stationary phase approximation, this leads to^45^:

3 $$I(\omega )\propto |\,\widetilde{f}(\omega ){|}^{2}{\omega }^{-4/3}{\{{{\rm{A}}{\rm{i}}}^{{\rm{{\prime} }}}[{(\omega /{\omega }_{\gamma })}^{2/3}]\}}^{2}$$

where Ai′ is the derivative of the Airy function of the first kind, ${\omega }_{\gamma }=\sqrt{8{\alpha }_{1}}{\gamma }^{3}$, and *γ* is the Lorentz factor at the time of emission $(\gamma =1/\sqrt{1-{v}^{2}/{c}^{2}})$. $\widetilde{f}(\omega )$ is the Fourier transform of the shape function *f* describing the finite width of the electron bunch. For *ω* ≪ *ω*_*γ*_ and an electron bunch much narrower than 1/*ω*, we recover the power law spectrum:

4 $$I(\omega )\propto {\omega }^{-4/3}$$

For this to be valid, *ω*/*ω*_*L*_ must also be sufficiently large that *t*/*T*_*L*_ is small in the expansions of *x*(*t*) and *j*_*y*_(*t*) and higher order terms can be safely neglected.

**Figure 7:**
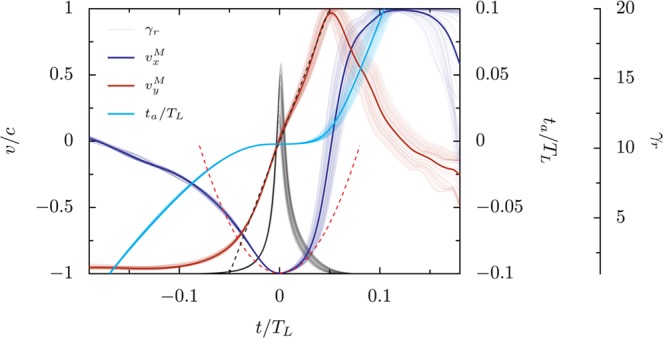
Velocity, Lorentz factor, and advanced time for emitting electron bunch at *a*_0_/*N* = 0.33, *a*_0_ = 100, and *θ* = 30°. The dashed lines show parabolic and linear fits to the relevant components of the velocity.

The mean and individual longitudinal and transverse velocities of an XUV-emitting electron bunch are shown in Fig. 7, along with the individual Lorentz factors in the direction of emission and the advanced time [*t*_*a*_ = *t* + *x*(*t*)/*c*] for each electron. To reach Eq. (3) we must fit the longitudinal velocity with a parabola and the transverse velocity with a line, as marked by the dashed curves. The width of the region where these approximate the underlying trajectories sets the lowest harmonic frequency which will follow Eq. (3); 4/3-scaling comes from the third-order approximation of *x* and it is in principle possible to reach shallower power laws for a single electron if higher-order terms cannot be neglected. However, under most observed conditions, the third order term in *x* dominates even for relatively large *t*/*T*_*L*_ (low-order harmonics) and the spectra produced by individual electrons tends to follow Eq. (3) with *f* = 1.

If the spectra associated with single electrons are well-predicted by Eq. (3) with $| \widetilde{f}(\omega ){| }^{2}=1$, deviations of the total reflected spectrum from this shape can be attributed to either the finite distribution in time and space of the electrons as they emit or the range in maximum Lorentz factors, and therefore *ω*_*γ*_, of the radiating electrons. Appropriate treatment of *f* allows the finite distribution of electrons to be included. The curves in Fig. 7 represent a selection of particles that reach the highest velocities, rather than all macroparticles in the simulation. These are the electrons that will dominate the emitted radiation, particularly at high frequencies, but the contribution of slower electrons to the total spectrum can result in relatively higher emission in low-order harmonics and a steeper power-law slope.

**Figure 8:**
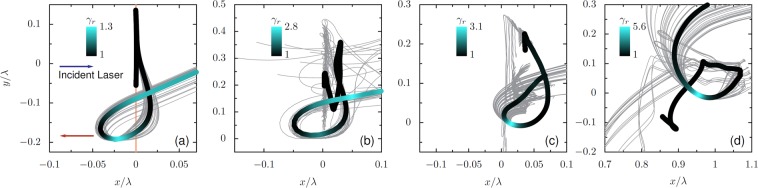
Synchrotron-like trajectories of emitting electron bunches across a range of conditions. Selected trajectories are marked in grey. The thick black-blue line shows the average trajectory, with the color indicating the Lorentz factor in the direction of specular reflection. For (**a**,**b**) this is along the *x* axis and for (**c**,**d**) the reflected direction is angled up by 30°. (**a**) Thin foil interaction with *D* = 2 nm, *N* = 500, *a*_0_ = 10, *θ* = 0°. (**b**) Trajectories extracted from a 2D PIC simulation for a thin foil target with *D* = 4 nm, *a*_0_ = 10, *N* = 500, and *θ* = 0°. (**c**) Semi-infinite interaction with *D* = *λ*/2, *a*_0_ = 40, *N* = 200, *θ* = 30°. (**d**) Interaction with a density gradient, with *L* = 0.1*λ*, *a*_0_ = 20 and *θ* = 30°. Synchrotron-like instantaneous motion appears under all these conditions around the time of emission.

The electron-bunch kinematics shown in Fig. 7 produce characteristic synchrotron-like trajectories in the *x*-*y* plane^14,46^. The key attribute of these trajectories is that velocity and acceleration are perpendicular when the electrons are traveling in the specular-reflection direction, a characteristic of synchrotron-like motion that can be found under many variations of the laser and plasma conditions. Figure 8 shows synchrotron-like trajectories from PIC simulations of a thin foil at normal incidence in one (a) and two (b) dimensions, (c) of a semi-infinite target at oblique incidence, and (d) of a finite plasma density gradient at oblique incidence. In the oblique incidence examples, the direction of emission is 30° above the *y* = 0 axis. In each of the subplots, the specular direction Lorentz factor peaks while the transverse acceleration is maximized, producing instantaneously circular trajectories. These characteristic synchrotron-like trajectories appear under a broad range of interaction conditions, so understanding their dynamics is critical to quantifying relativistic RHHG.

**Figure 9:**
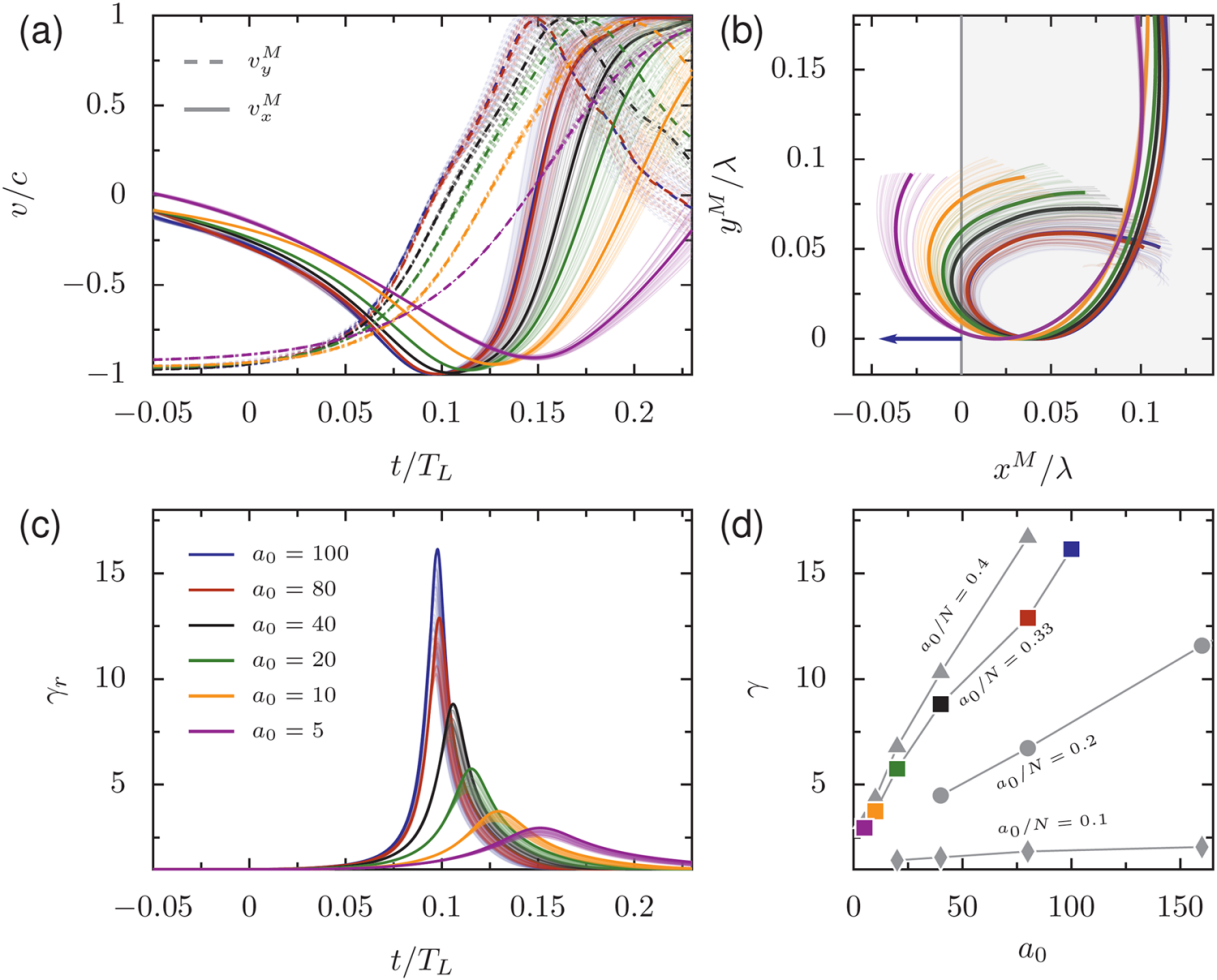
Kinematics of highest-*γ* emitting electrons: (**a**) the longitudinal (*v*_*x*_) and transverse (*v*_*y*_) velocities in the boosted reference frame, (**b**) the trajectories of the electrons in *x*-*y* space, and (**c**) the Lorentz factor of the electrons as a function of time. In (**b**), the gray region marks the ion density, and the blue arrow marks the direction of emission. For (**a,b**) the thick lines are the average values and thin lines are for individual particles. In (**c**) the thick lines mark the maximum *γ* factor. (**d**) The maximum Lorentz factor in the direction of emission for varied *a*_0_ and *a*_0_/*N*. Colored points correspond to those in (**c**). For fixed *a*_0_/*N*, *γ* is close to proportional to *a*_0_. For (**a–c**), *a*_0_/*N* = 0.33. For (**a–d**), *θ*_*L*_ = 30°.

Similarity theory suggests that in the limit *a*_0_ → *∞* at fixed *a*_0_/*N* the electron trajectories will approach an asymptote. Figure 9 shows the (a) electron bunch velocity components, (b) trajectories, (c) Lorentz factors for varied *a*_0_ at fixed *a*_0_/*N*, and (d) the maximum Lorentz factors over a range of *a*_0_ and *N*. From these plots it is apparent that the electron kinematics and trajectories for fixed *a*_0_/*N* approach a limit; the velocities and displacements for *a*_0_ = 80 and *a*_0_ = 100 are almost identical. This is in agreement with the collapse in Figs. 4 and 5b of *p*, which depends on lower-order harmonics and thus the shape of the electron trajectories over a relatively long time, to an asymptotic value in the same limit. For example, since the shape of the electron bunch velocity curves are close to identical for *a*_0_ = 80 and *a*_0_ = 100 over the interval 0.05 < *t*/*t*_*L*_ < 0.15, the tenth harmonic should be the same proportion of the fundamental for both.

However, the Lorentz factor of the electron bunch (*γ* = 1/$\sqrt{1-{v}^{2}/{c}^{2}}$ or, when calculated only for the velocity component in the direction of reflection, *γ*_*r*_ = 1/$\sqrt{1-{v}_{r}^{2}/{c}^{2}}$) does not collapse and instead scales linearly with *a*_0_ (Fig. 9c,d), which is consistent with the prediction from similarity theory that $\gamma \propto {S}^{{\alpha }_{S}}{a}_{0}$^53^. Note that because emission occurs when the velocity points in the specular reflection direction, *γ* = *γ*_*r*_ at the time of emission. To be consistent with the literature, where not otherwise noted the variable *γ* will indicate the value of the Lorentz factor at the emission time (which is generally the largest value of *γ*_*r*_). To be valid for moderate *a*_0_, we write this as:

5 $$\frac{\gamma -1}{{a}_{0}}\propto {\left(\frac{{a}_{0}}{N}\right)}^{-{\alpha }_{S}}$$

The cutoff frequency is dependent on *γ* so the efficiency of the highest frequencies may depend on *a*_0_ independent of *a*_0_/*N*, Both the ROM^10^ and CSE^45,46^ models predict scaling of the cutoff with *γ*^3^. The electron trajectories are therefore in agreement with the observation in Fig. 4 that *p* approaches an asymptotic limit for large *a*_0_, but the point at which the spectra deviate from *p* continues to vary with *a*_0_.

**Figure 10:**
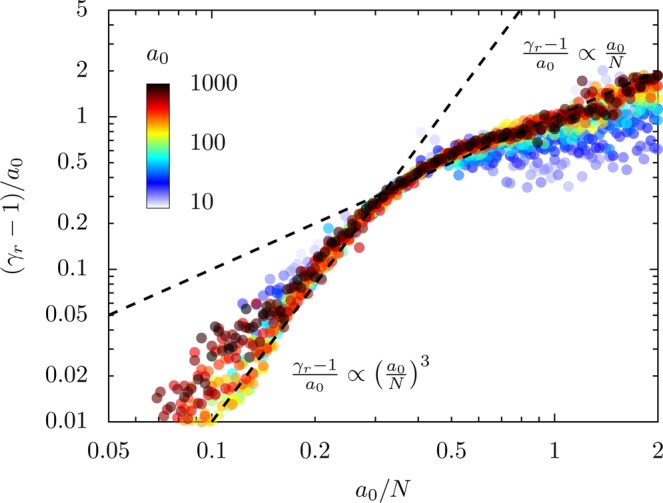
Peak Lorentz factor in the direction of emission for the emitting electron bunch for varied *a*_0_ and *N* at *θ*_*L*_ = 30°. Since *γ* − 1 ∝ *a*_0_, the normalized quantity $({\gamma }^{\max }-1)$/*a*_0_ collapses to a single curve *f*(*a*_0_/*N*) asymptotically for *a*_0_ → *∞*, with two distinct power-law regimes. For low *a*_0_/*N*, the Lorentz factor approximately scales with ${({a}_{0}/N)}^{3}$ and for *a*_0_/*N* > 0.3, corresponding to the initial onset of the relativistic transparency regime, it scales proportional to *a*_0_/*N*. Each point is extracted from a separate PIC simulation with a single-cycle driving laser.

Since we have shown that *γ* − 1 ∝ *a*_0_ at fixed *a*_0_/*N*, the final question is how (*γ* − 1)/*a*_0_ varies with *a*_0_/*N*. In Fig. 10 the normalized emitting-direction maximum Lorentz factor for electrons is plotted against *a*_0_/*N* for varied *a*_0_ and *N* at *θ*_*L*_ = 30°. The quantity (*γ* − 1)/*a*_0_ collapses to a single line with a double power-law shape, with (*γ* − 1)/*a*_0_ approximately proportional to ${({a}_{0}/N)}^{3}$ in the region *a*_0_/*N* < 0.3 and to *a*_0_/*N* in the region *a*_0_/*N* > 0.3. Note that due the transition of *α*_*s*_, other normalizations of *γ* will not produce a single collapsed curve. If the values of *γ* are compared to *I*_*a*_/*I*_*L*_ in Fig. 5, we can see that *γ* continuously increases with *a*_0_/*N* over the entirety of the optimal harmonic efficiency region, so the peak in attosecond pulse generation efficiency cannot be tied to a peak in *γ*. This suggests that an analysis that includes only *γ* in harmonic generation is missing key physics; in the next section we will address this gap by considering the spectral cutoff.

## The Harmonic Cutoff

To determine the efficiency of harmonic conversion, especially for x rays, we must know the highest frequency for which power-law scaling with *p* is valid. The spectral cutoff sets the limit of *p*-scaling and is thus critical for determining the x-ray generation efficiencies that can be reached. We have chosen here to define the observed cutoff frequency *ω*_*c*_ as the point where the spectrum has dropped below *ω*^−*p*^ by a factor of 1/*e*, in part because ${[{{\rm{Ai}}}^{{\prime} }(0)/{{\rm{Ai}}}^{{\prime} }(1)]}^{2}\approx 1$/*e*, so that when *ω* = *ω*_*γ*_, the value of *I*(*ω*) from Eq. (3) is 1/*e* times that from the  −4/3 scaling law. Therefore, if *ω*_*γ*_ governs the cutoff, *ω*_*γ*_ will approximately equal *ω*_*c*_.

We have previously established that *γ* ∝ *a*_0_ at fixed *a*_0_/*N*, and ${\omega }_{\gamma }=\sqrt{8{\alpha }_{1}}{\gamma }^{3}$, so for *a*_0_ ≫ 1:

6 $${\omega }_{\gamma }={a}_{0}^{3}\cdot f({a}_{0}/N,...)$$

The similarity-scaling of electron trajectories suggests that because *α*_1_ is derived from the shape of the electron velocity, it is a function of *a*_0_/*N* and does not strongly depend on *a*_0_ independently.

**Figure 11:**
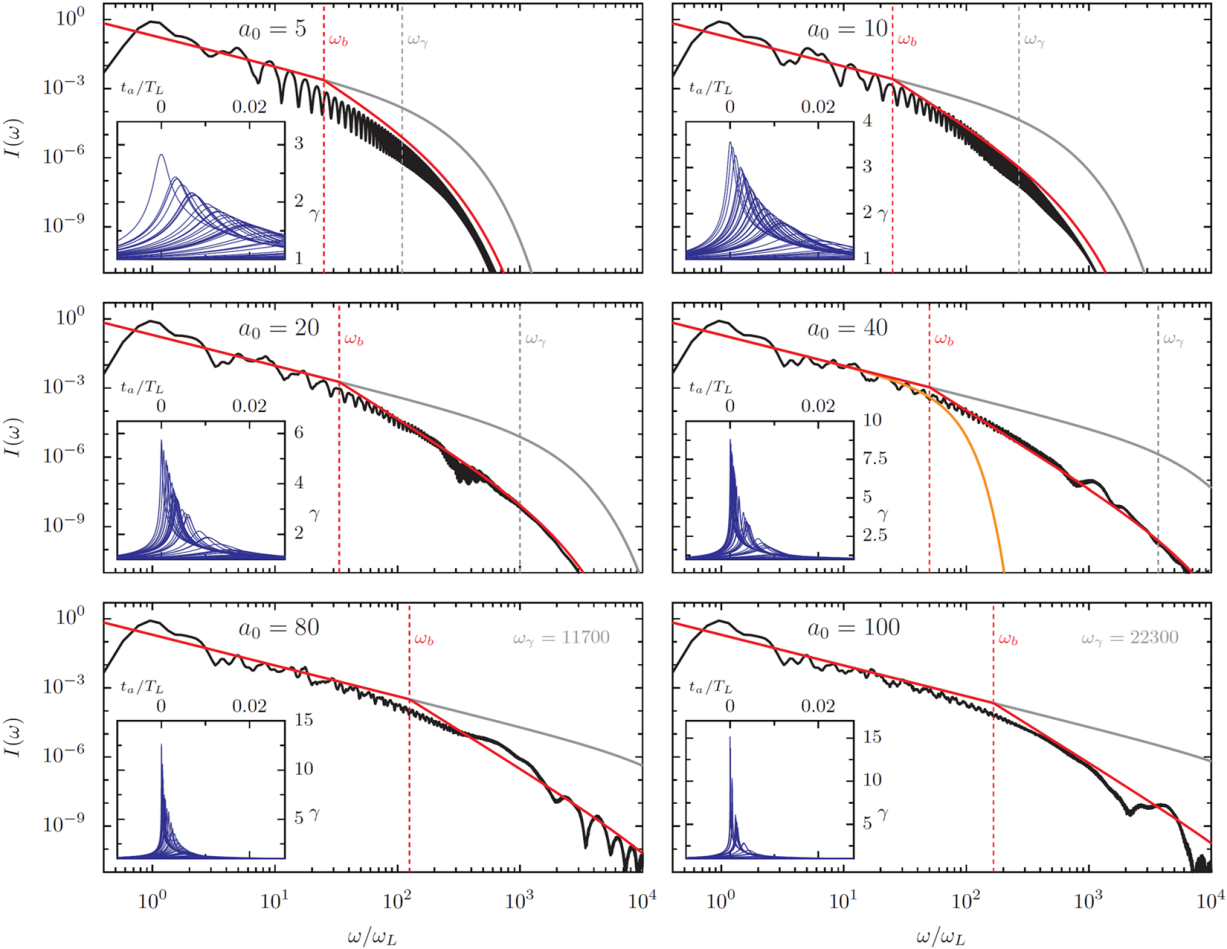
HHG spectra with CSE model fits and comparison to bunch width (*ω*_*b*_) and Lorentz factor (*ω*_*γ*_) cutoffs at *a*_0_/*N* = 0.33 and *θ* = 30°. Spectra (black) for *a*_0_ varied between 5 and 100 driven by single-cycle laser pulses. The CSE Airy-function spectrum neglecting bunch shape is shown in gray. In red, the analytic spectrum is corrected for the observed spread of emission times by assuming that only electrons within a half wavelength of the bunch leading edge emit constructively and that electron emission events are evenly distributed in time with a sharp initial edge. This last assumption is confirmed by the inset plots of electron *γ*-factor against the advanced time of the individual electrons, radiation from the fastest electrons arrives first, with minimal interference, after which the bunch slowly trails off. The inset plots contain a random selection of electron trajectories. The incoherent combination of higher-order frequencies results in an additional *ω*^−2^ factor in the spectral scaling above *ω*_*b*_ = (*T*_*L*_/2*τ*_*a*_)*ω*_0_. *ω*_*γ*_ is the cutoff calculated from the Lorentz factor: ${\omega }_{\gamma }=\sqrt{8{\alpha }_{1}}{\gamma }^{3}$. The bunch width is the time between the peak *γ*-factor of the first and last electrons to emit. For *a*_0_ = 40, an additional line (orange) shows the spectrum for a Gaussian emitting bunch shape, which leads to a factor $| \widetilde{f}(\omega ){| }^{2}=\exp [-{(\omega /{\omega }_{b})}^{2}]$ in Eq. (3)^45^.

Consider Fig. 11, where reflected spectra calculated for *a*_0_/*N* = 0.33, *θ* = 30°, and selected *a*_0_ between 5 and 100 are compared to Eq. (3) (gray line). We calculate the predicted cutoff *ω*_*γ*_ from the maximum *γ* of the emitting electron bunch observed in the PIC simulations and fitted *α*_1_ to the longitudinal velocity traces, which the insets show for randomly-selected particles as a function of the advanced time *t*_*a*_. The predicted cutoff *ω*_*γ*_ scales favorably with *a*_0_: at *a*_0_ = 100, we find *ω*_*γ*_ = 22300, implying that for *p* = 4/3 the process would produce 20 keV photons with 10^−6^ efficiency in each harmonic, or greater than 10^−3^ energy conversion efficiency to the spectral band 20–22 keV. However, the *a*_0_ = 100 spectrum actually begins to deviate from the  −4/3 law around *ω*/*ω*_*L*_ = 200, and the 10 keV efficiency is 10^−11^ per harmonic, five orders of magnitude below the optimistic power-law-*ω*_*γ*_ prediction. Though this still makes RHHG an extremely bright x-ray source, the substantial discrepancy requires explanation. The spectral cutoffs *ω*_*c*_ are much smaller than *ω*_*γ*_ in each of these plots, and, like the cutoffs in Fig. 4, appear to scale close to linearly with *a*_0_, not as ${a}_{0}^{3}$. Since *ω*_*γ*_ is often assumed to predict the maximum frequencies that can be efficiently generated, it is crucial to understand why RHHG spectra deviate from  −4/3 scaling at much lower frequencies.

The trajectory analysis in Fig. 7 substantiates Eqs. (1) and (2) for the average bunch trajectory, and it has been established elsewhere that Eq. (3) accurately predicts the spectra of individual high-*γ* electrons^58^, so it is initially surprising that *ω*_*γ*_ fails to match *ω*_*c*_ so dramatically. To address the discrepancy, we consider the width of the emitting electron bunch by accurately including the shape factor term $| \widetilde{f}(\omega ){| }^{2}$ that appears in the original formulation of Eq. (3). There are two important differences with how we treat the emitting electrons in comparison to the previous literature. First, we must consider the emission width as a function of the advanced time, not just approximate the width of the electron nanobunch in space at a fixed laboratory time. Since the electrons move relativistically and emission time in the laboratory frame varies with position in the bunch, the dependence of advanced time (*t*_*a*_) on time (*t*) is complicated. Second, the distribution is not Gaussian; the electron emission distribution has a sharp leading edge, so a better approximation is the Heaviside step function, resulting in a power-law decay rather than an exponential cutoff at *ω*_*b*_. This sharp leading edge can be seen in the insets of Fig. 11, which show the individual particle Lorentz factors as a function of advanced time. The distribution of emission events – which is related to, but not exactly the same as, any instantaneous electron density profile – more directly affects the total emitted radiation than the electron number density. The number density of electrons is proportional to the density of drawn lines in these plots. In each plot, emission from the fastest electrons arrives at the observation point first. After the peak, the rate of emission events remains relatively constant as the maximum Lorentz factor of the involved electrons slowly decays, which is due to electrons further from the surface feeling weaker fields. The step function is an appropriate approximation because the rate of emission events does not slowly ramp up before the peak arrives; in each of these plots no emission peak is produced before *t*_*A*_ = 0. The difference between the orange line (Gaussian) and red line (step function) spectra for *a*_0_ = 40 in Fig. 11 illustrates the enormous impact the actual distribution of charge in the electron nanobunch has on the high-frequency components of the spectrum.

The distributions of peak *γ*-factors, which indicate when the highest frequency radiation is emitted by each electron, can have widths that are a substantial fraction of the laser period. For a given harmonic (*n*) with frequency *ω*_*n*_ = *nω*_*L*_, only electrons within about half a harmonic period (*T*_*n*_ = *T*_*L*_/*n*) of the leading edge will contribute. The advanced time electron bunch width *τ*_*a*_ therefore sets a frequency cutoff *ω*_*b*_ approximated as:

7 $$\frac{{\omega }_{b}}{{\omega }_{L}}\approx \frac{{T}_{L}}{2{\tau }_{a}}$$

At *a*_0_ = 10, for example, the emission lasts for around 2% of the laser period, which implies that all of the electrons in the bunch only contribute constructively to the first 25 harmonics. From a physical perspective, if we assume that the electron emission times are uniformly distributed after the leading edge, the condition that only those within *T*_*n*_/2 constructively add means the fraction of the electron bunch that contributes to the emission of frequency *ω*_*n*_ scales as 1/*n*. For coherent radiation, the radiated power will be proportional to 1/*n*^2^, or, equivalently, ${\omega }_{n}^{-2}$. Mathematically, the square of the Fourier transform of the Heaviside step function will lead to an additional *ω*^−2^ factor for *ω* > *ω*_*b*_, i.e.:

8 $$I(\omega )\approx \{\begin{array}{cc}{C}_{1}{\omega }^{-4/3}{\{{{\rm{A}}{\rm{i}}}^{{\rm{{\prime} }}}[{(\omega /{\omega }_{\gamma })}^{2/3}]\}}^{2} & \,{\rm{i}}{\rm{f}}\,\omega  < {\omega }_{b},\\ \\ {C}_{2}{\omega }^{-10/3}{\{{{\rm{A}}{\rm{i}}}^{{\rm{{\prime} }}}[{(\omega /{\omega }_{\gamma })}^{2/3}]\}}^{2} & \,{\rm{i}}{\rm{f}}\,\omega \ge {\omega }_{b}.\end{array}$$

where *C*_1_ is a constant and ${C}_{2}={\omega }_{b}^{2}{C}_{1}$. We apply this correction to the spectra in Fig. 11 to plot the red curves, using detailed particle trajectories to calculate *τ*_*a*_ and *ω*_*b*_. The corrected predictions much more closely approximate the actual spectra, and the correct treatment of the bunch width becomes critically important for keV photon energies. Given that much of the detailed structure of the electron bunches has been approximated away without much justification, the analytic spectra match the PIC results remarkably well. Note that although the analytic fit has a sharp angle between the two scaling regimes, we expect both experimental and simulation spectra to exhibit a smoother transition because the onset of destructive interference is gradual.

**Figure 12:**
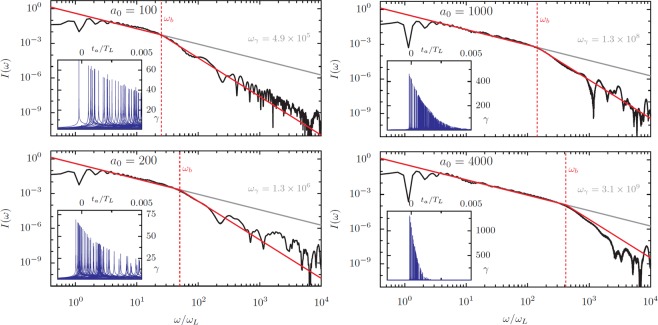
HHG Spectra with CSE model fits and comparison to bunch width and Lorentz factor cutoffs at *a*_0_/*N* = 1.1 and *θ* = 45°. As in Fig. 11, the PIC-derived spectra are compared to analytic fits based on the CSE model with and without bunch-width correction, with the cutoffs associated with both the bunch width and the electron Lorentz factor marked. Inset: the electron Lorentz factors in the emission direction as a function of the advanced time. Note that for these conditions the width in *γ*_*r*_ for an individual electron is even narrower in comparison to the total width of the bunch than for the trajectories in Fig. 11.

The importance of *ω*_*b*_ extends further than the single value of *a*_0_/*N* presented in Fig. 11 and is starkly apparent in Fig. 12, where spectra and electron *γ*-factors are shown for selected *a*_0_ at *a*_0_/*N* = 1.1 and *θ*_*L*_ = 45°. Higher values of *a*_0_/*N* produce higher *γ* factors (see Fig. 10), and *a*_0_/*N* = 1.1 gives *γ* ≈ 0.3*a*_0_, producing *ω*_*γ*_ at MeV levels for *a*_0_ = 100 and at 5 GeV for the, admittedly unrealistic, *a*_0_ = 4000. However, *ω*_*b*_ is even smaller for this larger value of *a*_0_/*N*, leading to bunch-width-dependent cutoffs at much lower frequencies. Note that for this *a*_0_/*N* the fundamental and lower-order harmonics are not strongly generated, so the first few harmonics appear to scale above the  −4/3 power law. The advanced time *γ*-factor shapes for individual electrons are much narrower than the total time over which the emission arrives, which can be viewed as the physical interpretation of *ω*_*γ*_ ≫ *ω*_*b*_.

**Figure 13:**
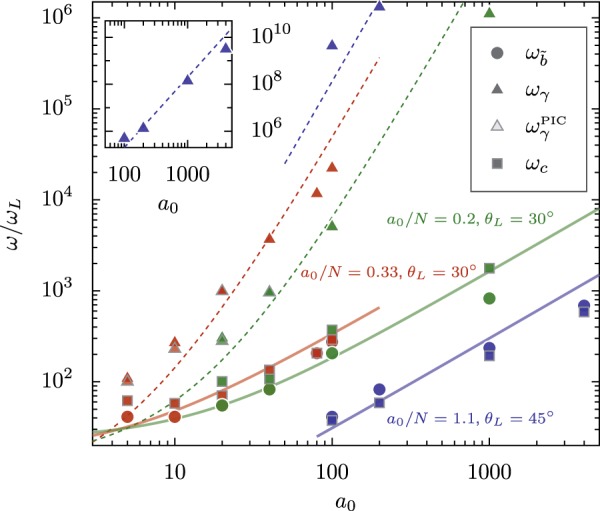
Variation with *a*_0_ of the observed spectral cutoff (*ω*_*c*_), the modified bunch-width-dependent cutoff (${\omega }_{\widetilde{b}}$), the Lorentz-factor-dependent cutoff (*ω*_*γ*_) and the Lorentz factor cutoff observed in the PIC spectra (${\omega }_{\gamma }^{{\rm{PIC}}}$) at *a*_0_/*N* = 0.33 (red) and *a*_0_/*N* = 0.2 (green) with *θ*_*L*_ = 30° and *a*_0_/*N* = 1.1 with *θ*_*L*_ = 45° (blue). The predicted *γ*^3^ dependence of *ω*_*γ*_ is represented by the dashed lines, calculated from $\sqrt{8{\alpha }_{1}}{({C}_{\gamma }{a}_{0}+1)}^{3}$, where both *C*_*γ*_ and *α*_1_ are estimated from the scaling of *γ* with *a*_0_/*N*. The discrepancy between the dashed line and *ω*_*γ*_ points can be tied to *γ* not being exactly proportional to *a*_0_; the values of *ω*_*γ*_ found from the electron trajectories and ${\omega }_{\gamma }^{{\rm{PIC}}}$ found directly from the spectra are almost indistinguishable. The solid lines are approximations based on *ω*_*b*_ ∝ *γ*. Inset: *ω*_*γ*_ for large values of *a*_0_.

To present a more general picture of how the different measures of cutoff vary, the variation in *ω*_*b*_, *ω*_*γ*_ and *ω*_*c*_ with *a*_0_ is presented in Fig. 13 for *a*_0_/*N* = 0.33 (red) and *a*_0_/*N* = 0.2 (green) at *θ*_*L*_ = 30° and *a*_0_/*N* = 1.1 at *θ*_*L*_ = 45° (blue), which are representative of the spectral behavior in the efficient regime of harmonic generation. To allow comparison of *ω*_*c*_ and *ω*_*b*_ we define ${\omega }_{\widetilde{b}}=\sqrt{e}\cdot {\omega }_{b}$, so that ${\omega }_{\widetilde{b}}$ is the point where the *ω*_*b*_-corrected spectrum has dropped by 1/*e* below the original *p* scaling. If the deviation from *p*-scaling is determined by *ω*_*γ*_, we should have *ω*_*c*_ ≈ *ω*_*γ*_. Instead, we have ${\omega }_{c}\approx {\omega }_{\widetilde{b}}$ for the full range of representative conditions shown. Although *ω*_*γ*_ reasonably well follows $\sqrt{8{\alpha }_{1}}{({C}_{\gamma }{a}_{0}+1)}^{3}$ for *C*_*γ*_ found from Fig. 10 and *α*_1_ found from the average velocity (dashed lines), both ${\omega }_{\widetilde{b}}$ and *ω*_*c*_ increase much less rapidly, with the solid lines linearly proportional to *γ*. The linear *γ* scaling of *ω*_*b*_ is simply an approximate empirical fit to this data and a derived relationship between the two may point to a slightly different functional relationship. The linear fit may also not be general. We can, however, say with some confidence that neither *ω*_*b*_ nor *ω*_*c*_ is proportional to *γ*^3^. In plots where *ω*_*γ*_ lies below *ω*/*ω*_*L*_ = 1000 the exponential cutoff can be identified directly from PIC spectra in addition to the *ω*_*b*_ cutoff; for each of these simulations, *ω*_*γ*_ calculated from the electron *γ*-factors closely agrees with ${\omega }_{\gamma }^{{\rm{PIC}}}$ found by direct fitting to the observed spectrum. Note that the Doppler mirror cutoff^8^ at 4*γ*^2^ does not appear as a feature in any of the spectra which contribute to this plot.

**Figure 14:**
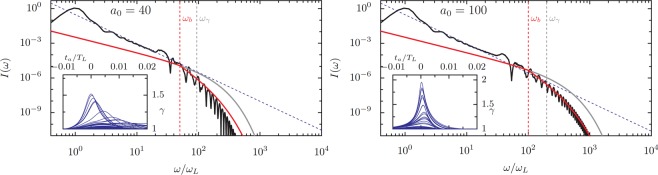
HHG spectra with CSE model fits and comparison to bunch width and Lorentz factor cutoffs at *a*_0_/*N* = 0.1 and *θ* = 30°. In this lower efficiency regime, the slope *p* is not close to 4/3. The difference between the 4/3 power law scaling (red) and the actual scaling (dashed blue) results from the substantial contribution in these spectra of electrons which have low maximum velocities compared to the peak of the electron bunch. Here, *ω*_*b*_ ≈ *ω*_*γ*_.

We must also consider whether these relationships hold under less-efficient conditions, where *p* is substantially larger than 4/3. In Fig. 14, where *θ* = 30° and *a*_0_/*N* = 0.1, *p* lies around 2.5 for both *a*_0_ = 40 and *a*_0_ = 100. For these parameters, a significant fraction of the electrons which contribute to the reflected radiation do not reach the peak Lorentz factor associated with the overall interaction. Since the *γ* achieved by each electron determines the highest frequencies it will emit, the number of electrons which contribute to a particular harmonic decreases for higher harmonic. This causes a difference between the 4/3 scaling of the CSE model and the steeper scaling of the observed spectrum for a less ideal ratio of *a*_0_ to *N*. In these interactions *ω*_*b*_ and *ω*_*γ*_ are comparable, so although the spectral fit for the bunch-width correction appears to better match the data the difference is relatively small.

**Figure 15:**
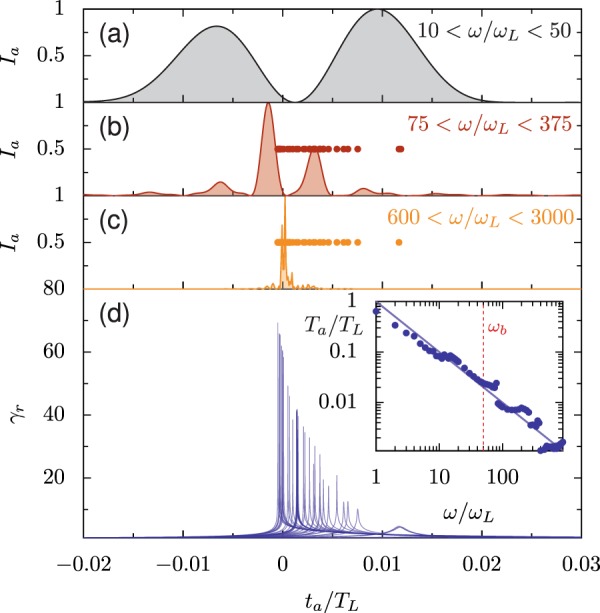
Instantaneous intensity of reflected radiation in the lab frame after filtering to include only frequencies in the ranges (**a**) 10 < *ω*/*ω*_*L*_ < 50, (**b**) 75 < *ω*/*ω*_*L*_ < 375, and (**c**) 600 < *ω*/*ω*_*L*_ < 3000 and (**d**) the corresponding *γ*-factors in the advanced time. Inset: the attosecond pulse duration *T*_*a*_ (full-width-half-maximum of the envelope) for varied *ω*_*LF*_ where *ω*_*UF*_ = 5*ω*_*LF*_. The line marks *T*_*a*_/*T*_*L*_ = 1/(*ω*_*LF*_/*ω*_0_), with the agreement between the line and points suggesting the attosecond pulse duration depends directly on the wavelength of its component light. In (**b**) and (**c**), the circles indicate the emitting time of all electron trajectories where *ω*_*γ*_ > *ω*_*LF*_. Without destructive interference between electrons, we would expect the attosecond pulse in (**c**) to have a duration on the scale of the distribution of circles. These plots are taken at *a*_0_/*N* = 1.1, *a*_0_ = 200, and *θ*_*L*_ = 45° and correspond to that shown in Fig. 12c. For frequencies higher than 1000*ω*_*L*_ numerical dispersion of the propagating radiation has a measurable widening effect on the duration of the attosecond pulse. Note that for (**a**–**c**) we consider light passing the observation point, so the advanced time is equivalent to the lab frame.

The interpretation of the steep power-law-like behavior of spectra in the regime *ω* > *ω*_*b*_ as due to destructive interference of electrons emitting more than *λ*_*n*_/2*c* after the sharp leading edge can be checked by filtering the reflected radiation and examining it in the time domain. Figure 15 shows the intensity of reflected radiation as a function of time for three different filters. Even for the lowest frequency filter (10 < *ω*/*ω*_*L*_ < 50), we have created an attosecond pulse: the approximate duration produced by an 800 nm driver is 100 as. For 600 < *ω*/*ω*_*L*_ < 3000, the process produces an attosecond pulse with 4 as duration. For filters with *ω*_*LF*_ > *ω*_*b*_, the attosecond pulse is much shorter than the duration of the emitting electron bunch in the advanced time (Fig. 15d); the circles in (b) and (c) indicate the times at which electrons with individual *ω*_*γ*_ greater than *ω*_*LF*_ reach their maximum *γ*_*r*_. Without destructive interference between electrons after the leading edge of the bunch, this would be the width of the attosecond pulse for this filter. The inset in Fig. 15d shows the full-width-half-maximum duration (*T*_*a*_) of the attosecond pulse as a function of the filter frequency *ω*_*LF*_, where *ω*_*UF*_ = 5*ω*_*LF*_, which follows the scaling *T*_*a*_/*T*_*L*_ = *ω*_*L*_/*ω*_*LF*_ closely. *ω*_*LF*_ and *ω*_*UF*_ define the filter passband; the filter is transparent for *ω*_*LF*_ < *ω* < *ω*_*UF*_. Therefore, although the efficiency of harmonic generation drops for *ω* > *ω*_*b*_, the duration of the attosecond pulses that are produced continues to shorten.

## Beyond Single-Cycle Semi-Infinite Interactions

Although the use of single-cycle interactions on plasma targets with steep density transitions between vacuum and solid density allows study of the underlying RHHG dynamics, real interactions often involve multi-cycle laser pulse on targets which have developed finite exponential density gradients. We consider here how both gradients and multiple-cycle pulses affect the scaling of the reflected spectrum.

### Finite plasma density gradients

Over a picosecond to nanosecond timescale after ionization of a solid density target by a laser pulse, the plasma formed at the surface will expand outwards self-similarly, forming an exponential plasma density gradient [$N(x)={N}_{\max }{e}^{x/L}$]^59,60^. For relativistic surface interactions, even prepulses orders-of-magnitude weaker than the main pulse arriving tens-to-hundreds of picoseconds earlier can perturb the target enough to create a substantial plasma density gradient. The effect of pre-plasma gradients on RHHG has been studied^28,29^, and though there is some disagreement over the optimal scale length, most experimental and theoretical results point to enhanced harmonic generation for *L*/*λ* up to a few tenths of the laser wavelength. We must therefore establish how the analysis developed for plasma targets with sharp edges extends to the plasma density gradient case: here we show that (1) small-to-moderate gradients increase efficiency for *a*_0_/${N}_{\max }\ll 1$, (2) that optimal gradient scale lengths have efficiencies similar to semi-infinite targets with ideal *a*_0_/*N*, and (3) that for longer gradients it becomes more difficult to create a narrow emitting electron bunch.

**Figure 16:**
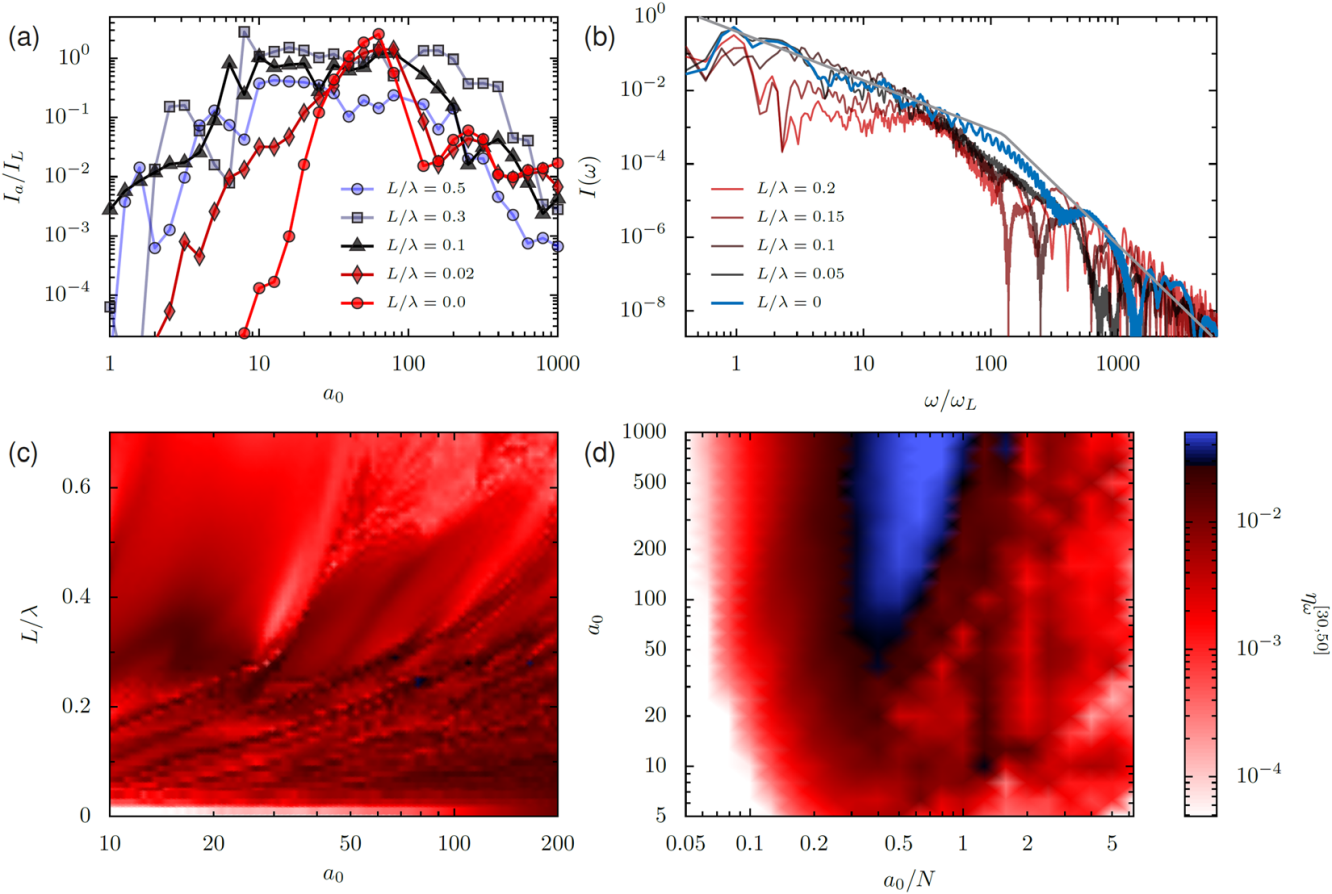
(**a**) Attosecond pulse generation efficiency for varied *a*_0_ and gradient scale length *L*. The plasma target has a maximum density of *N* = 200, so for *a*_0_ > 100, *a*_0_/*N* > 0.5 and the interaction enters the less-efficient transparency regime. *θ*_*L*_ = 30°. The filter applied to the reflected field selects 20 < *ω*/*ω*_*L*_ < 100. (**b**) Spectra produced for *a*_0_ = 100 and varied *L*, with *N* = 200 in the *L* = 0 case, and ${N}_{\max }=500$ in the finite gradient cases. The gray solid line shows the bunch-width-corrected spectral prediction for the no-gradient case. For all, *θ*_*L*_ = 45°. (**c,d**) Harmonic frequency conversion *η*_*ω*_ to the interval 30 < *ω*/*ω*_*L*_ < 50 for (**c**) a finite exponential gradient scale length *L* and (**d**) varied *a*_0_/*N* without a gradient. The color scale is set so that blue regions correspond to efficiencies greater than 50% of that produced by  −4/3 scaling. For (**c,d**), *θ*_*L*_ = 30°.

Let us first examine the effect of a gradient on attosecond pulse generation efficiency. In Fig. 16a, the ratio of attosecond intensity to incident intensity is plotted for a range of *a*_0_ values and selected *L*/*λ* between 0 and 0.5. The target maximum density is ${N}_{\max }=200$ and *θ*_*L*_ = 30°. For *L*/*λ* = 0, this means *a*_0_/*N* = *a*_0_/200 and the peak efficiency of *I*_*a*_/*I*_*L*_ ≈ 30 occurs around *a*_0_ = 70. The attosecond pulses are filtered from the reflected field in the interval 20 < *ω*/*ω*_*L*_ < 100. As the gradient is increased from 0 to *L*/*λ* = 0.1, the efficiency increases for *a*_0_/${N}_{\max } < 0.2$, which can be attributed to the interaction happening at a much lower density (*N* ≈ *a*_0_) than *N* = 200. Under these conditions, for *L*/*λ* > 0.1 the efficiency does not continue to increase; for gradients larger than 0.5*λ* the attosecond pulse become weaker. In the regime *a*_0_ > 100 the gradient has little effect, since the interaction is in the transparency regime for the maximum plasma density. These simulations are configured so that the gradient decreases exponentially to a minimum value of 0.01*n*_*c*_, where it drops to zero. Weighted particles are used to provide adequate resolution across the entire range of densities with reasonable computational expense.

The key point of Fig. 16a is that although a small (*L*/*λ* ≈ 0.1) gradient can improve RHHG efficiency by orders of magnitude for *a*_0_/*N* < 0.2 – which is an important regime experimentally – the peak efficiencies reached with a gradient are similar to or less than the maximum efficiencies reached for the optimal values of *a*_0_/*N* without a gradient. In this sense, gradients are simply a way of correcting target densities that are too high for currently reachable laser intensities. This, along with the observation that electron trajectories are similar for both efficient flat targets and gradients (Fig. 8), suggests that the corrected synchrotron model is also an adequate method to treat targets with moderate gradients.

If we compare the reflected spectra produced by targets with gradient scale lengths in the interval 0 ≤ *L*/*λ* ≤ 0.2 (Fig. 16b), we find that where the zero-gradient case is close to optimum (*a*_0_/*N* = 0.4), the gradient does not substantially change RHHG efficiency. For larger gradients, the reflectivity in the interval 2 < *ω*/*ω*_*L*_ < 30 drops, which flattens the apparent initial scaling law, but does not increase ${\eta }_{\omega }^{[n]}$ for *n* > 30, where ${\eta }_{\omega }^{[n]}$ is the fraction of incident energy reflected in harmonic *n* and ${\eta }_{\omega }^{[{n}_{1},{n}_{2}]}$ is the fraction of energy in the between harmonics *n*_1_ and *n*_2_, inclusive. In all finite-gradient cases significant modulations appear in the spectrum for higher frequencies, indicating complex plasma dynamics. These modulations produce significant apparent fluctuations in efficiency for particular intervals of the harmonic spectrum, but the large-scale behavior of each of the spectra shown here is a decrease at a similar rate.

To consider a broader range of parameters, we plot ${\eta }_{\omega }^{[30,50]}$ for both varied gradient scale length with ${N}_{\max }=1000$ (Fig. 16c) and varied *a*_0_/*N* without a gradient (Fig. 16d). The color scheme for both plots is chosen so that blue regions represent efficiencies greater than 50% of those predicted by the  −4/3 power law. Although the zero-gradient case reaches its optimum around *a*_0_/*N* ≈ 0.5 and *a*_0_ > 100, the finite gradient case is restricted to lower efficiencies, even for *a*_0_ up to 200. This suggests that although a gradient is useful for increasing efficiency for moderate intensity lasers, the ideal condition for RHHG is a semi-infinite target with density appropriately balanced to the laser field strength.

**Figure 17:**
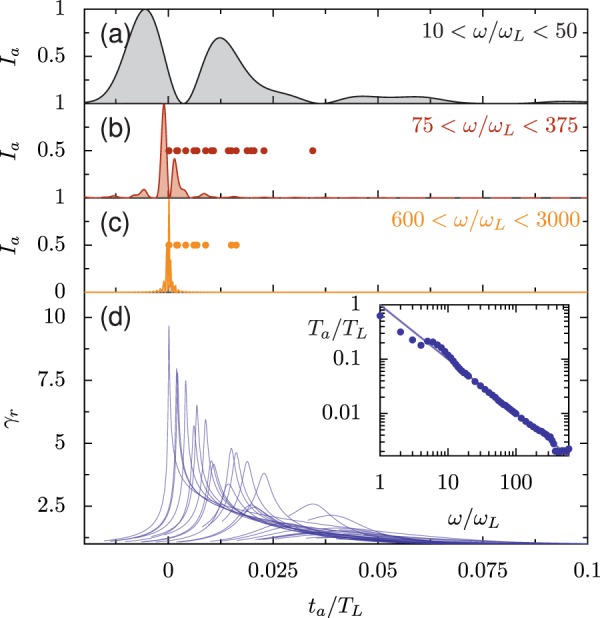
Intensity of reflected radiation after filtering to include only frequencies in the ranges (**a**) 10 < *ω*/*ω*_*L*_ < 50, (**b**) 75 < *ω*/*ω*_*L*_ < 375, and (**c**) 600 < *ω*/*ω*_*L*_ < 3000 and (**d**) the corresponding *γ*_*r*_ in the advanced time, for *L*/*λ* = 0.1, *a*_0_ = 20, and *θ*_*L*_ = 45°. ${N}_{\max }=1000$. Inset: the attosecond pulse duration *T*_*a*_ (full-width-half-maximum of the envelope) for varied *ω*_*LF*_ where *ω*_*UF*_ = 5*ω*_*LF*_. The line marks *T*_*a*_/*T*_*L*_ = 1/(*ω*_*LF*_/*ω*_0_). In (**b**) and (**c**), the circles indicate the emitting time of all electron trajectories where *ω*_*γ*_ > *ω*_*LF*_.

The trajectories of emitting electron bunches in finite gradients are similar to those for a high-efficiency flat target: as shown in Fig. 17, the width of the emitting electron bunch in the advanced time frame is much longer than the attosecond pulses that are produced for high frequencies. The destructive interference in the trailing edge of the electron bunch means that attosecond pulses are still produced, but the harmonic conversion efficiency drops below the ideal power-law scaling. Provided the gradient is reasonably short (*L*/*λ* < 0.2), the corrected synchrotron emission model is still applicable. The most efficient gradients are those with *L*/*λ* < 0.2, so the breakdown of the synchrotron model for very long gradients is less important for high-order harmonic generation.

### Multi-cycle driving pulses

The emphasis on single-cycle driving pulses in the previous sections has been justified by the fact that the frequency conversion process is intrinsically sub-cycle; the emission occurs within a fraction of the driving laser period. Here we briefly suggest how the single-cycle analysis extends to multi-cycle pulses, arguing that a long driving laser can be represented as a sequence of single cycle interactions provided that the initial conditions of each interaction can be treated appropriately.

The carrier envelope phase (CEP), represented here by *ϕ*, describes the alignment of the envelope and underlying phase. We take the convention *ϕ* = 0 for a sine-like pulse. Since the generation of attosecond pulses is associated with transitions of the laser electric field during particular optical half-cycles^15^, we can assign each attosecond pulse a phase *ϕ*_*a*_ based on the CEP of the pulse that produced it and the optical cycle from which it was generated. For example, an attosecond pulse generated during the central optical cycle for a *ϕ* = 0 incident beam has phase *ϕ*_*a*_ = 0 and additional pulses will be generated during different cycles of the same interaction with *ϕ*_a_ = *nπ* for normal incidence. We can in general write *ϕ*_a_ = *πn* + *ϕ*, noting that for oblique incidence, attosecond pulses at odd values of *n* will be suppressed, a similar role to that fulfilled by the differently-defined *ψ*_*g*_ used by Ma *et al*.^61^.

**Figure 18:**
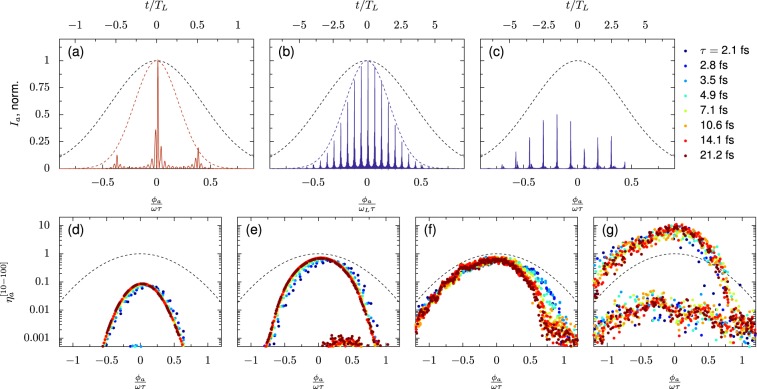
Dependence of attosecond pulse intensity (*I*_*a*_∕*I*_*L*_) on *τ* and *ϕ* at *a*_0_ = 100, *N* = 1000, and *θ*_*L*_ = 0°. (**a**) Attosecond pulse train for *τ* = 3.5 fs and (**b**) *τ* = 21.2 fs incident Gaussian pulses, plotted with incident intensity envelope (black dashed lines) and predicted attosecond pulse train envelope (red/blue dashed lines) for 10 < *ω*/*ω*_*L*_ < 100 based on *a*_0_/*N* scaling (*p* = 6.6) found for single-cycle pulses. (**c**) Dependence of attosecond pulse intensity (*I*_*a*_/*I*_*L*_) on *τ* and *ϕ* at *a*_0_ = 20, *N* = 80, and *θ*_*L*_ = 30°. (**a**) Attosecond pulse train generated by incident pulse at *τ* = 21.2 fs, and incident pulse intensity envelope (black dashed line). (**d–g**) Intensity of attosecond pulse trains produced by relativistic high harmonic generation (*a*_0_ = 100, *N* = 1000), for varied carrier envelope phase and pulse envelope intensity FWHM (*τ*), at (**a**) *θ*_*L*_ = 15° and *L*/*λ* = 0, (**b**) *θ*_*L*_ = 45° and *L*/*λ* = 0, (**c**) *θ*_*L*_ = 0° and *L*/*λ* = 0.05, and (**d**) *θ*_*L*_ = 45° and *L*/*λ* = 0.01 The attosecond pulses are calculated by filtering the reflected signal to the range 10 < *ω*/*ω*_*L*_ < 100. The *x*-axis is the attosecond phase normalized by the pulse duration and the *y*-axis is the attosecond pulse intensity normalized by the maximum incident laser intensity. The dashed lines indicate the intensity envelope of the incident signal; in these units the FWHM of the incident pulse (*τ*) is 1.

In the limit of short driving pulses (full-width-half-maximum duration *τ*), where the parasitic effects of preceding cycles can be neglected, the strength an emitted attosecond pulse will depend on the effective normalized potential ${\tilde{a}}$ during the optical half-cycle which leads to its emission. A Gaussian incident laser [${\tilde{a}} (t)={a}_{0}\exp \{-b{\left(t/\tau \right)}^{2}\}$ where $b=2{\rm{ln}}\,2$] produces an attosecond pulse with phase *ϕ*_*a*_ has ${\tilde{a}} ({\phi }_{a})={a}_{0}\exp \{-b{\left({\phi }_{a}/{\omega }_{L}\tau \right)}^{2}\}$. For isolated interactions with steep surfaces, we may often write – as can be seen in Fig. 5a– *I*_*a*_/${I}_{L}\propto {({a}_{0}/N)}^{q}$ where ${I}_{L}\propto {a}_{0}^{2}$, so that for a Gaussian driving laser the attosecond pulse intensity scales with *a*_0_, *ϕ*_*a*_ and *τ* as ${I}_{a}({\phi }_{a})\propto {[ {\tilde{a}} ({\phi }_{a})]}^{q+2}={a}_{0}^{q+2}\exp \{-b(q+2){\left({\phi }_{a}/{\omega }_{L}\tau \right)}^{2}\}$. This equation predicts the attosecond pulse train shapes in Fig. 18a,b, where the relative intensities of attosecond pulses are presented for  −0.8 < *t*/*τ* < 0.8. In Fig. 18a,b, the attosecond pulse trains produced by *τ* = 3.5 fs and *τ* = 21.2 fs incident pulses agree with the predicted attosecond pulse envelope (red and blue dashed lines), based on *q* = − 6.6 calculated from *a*_0_/*N* scaling for single-cycle pulses under these conditions.

Figure 18d  summarizes attosecond pulse intensities calculated for a range of incident *τ* and *ϕ* at *a*_0_ = 100, *N* = 1000, and *θ*_*L*_ = 15°, showing that for these parameters the attosecond pulse intensity depends only on position under the envelope *ϕ*_*a*_/*ω*_*L*_*τ*, and that the relative strength of pulses in the train matches the expectation from the single-cycle scaling. In Fig.18e–g the attosecond pulse intensity (*I*_*a*_/*I*_*L*_) is plotted against the envelope position *ϕ*_*a*_/*ω*_*L*_*τ* for varied *θ*_*L*_ and *L*/*λ*, showing a reasonable collapse of *η*_*a*_ to a single function of *ϕ*_*a*_/*ω*_*L*_*τ* for varied *τ* across a range of conditions. The second line for *L*/*λ* = 0.1 and *θ*_*L*_ = 45° are from attosecond pulses emitted on alternative half-cycles from the main pulse.

Generation efficiency becomes more complicated for multi-cylce pulses under the most efficient conditions, where, for example at *a*_0_/*N* = 0.25, the maximum attosecond pulse intensity decreases for longer pulse duration (Fig. 18c). Since intensities for different values of *τ* coincide at early times on our normalized scale, the decrease in maximum intensity for longer *τ* can be attributed to the parasitic effect of strong early pulses on the achievable intensity in later cycles, through disruption of the plasma surface. This effect is not observed for smaller values of *a*_0_/*N* because the plasma response time is much faster than the laser period, so the disruption due to the generation of an attosecond pulse is damped before the next pulse arrives. In contrast, at higher *a*_0_/*N*, the relativistic plasma frequency is closer to the laser frequency and plasma disruption may persist for a full optical cycle.

The total spectrum will be the sum of the spectrum contributions the individual pulses. In all multi-cycle pulse cases, a range of intensities under the envelope drive different attosecond pulses, so the individual spectral contributions vary under the envelope. The total spectrum can therefore appear more complex, even if the individual attosecond pulse generation effects are well described by an analytic CSE model.

## Conclusion

In this article we have presented results from a large number of particle-in-cell simulations, focusing on how the reflected high-order harmonic spectra varies with plasma density, laser intensity, and angle of incidence, and we have explained key features in terms of a bunch-width corrected coherent synchrotron emission model. Specifically, we have shown that under most conditions the energy spectrum decays as a power of frequency with exponent  −*p* up to a rolloff set by the width of the emitting electron bunch. The spectrum then approximately decreases as *ω*^−*p*−2^, often with substantial modulation due to the interference between different parts of the electron bunch, until an exponential falloff at a frequency proportional to the cube of the electron Lorentz factor. The initial power-law exponent *p* appears limited to around 4/3, the prediction of the CSE model, so the maximum harmonic peak powers reachable in the 0.1-10 keV range are likely $0.33{(\omega /{\omega }_{L})}^{-4/3}{P}_{0}$ in each harmonic, where *P*_0_ is the initial power and the 0.33 factor arises from normalizing the total reflected power to the incident power. for a 10-PW driver at 800 nm, this leads to 13 TW/harmonic at 100 eV, 0.5 TW/harmonic at 1 keV, and 25 GW/harmonic at 10 keV.

Outside of the most efficient conditions, we have found that *p* is a continuous function of *a*_0_ and *N* which approaches an asymptotic limit for *a*_0_ → *∞* at fixed *a*_0_/*N* and decreases monotonically for *a*_0_/*N* < 0.5. The bunch-width cutoff *ω*_*b*_ normally lies at smaller frequencies than the Lorentz-factor dependent cutoff *ω*_*γ*_, leading to a steep power-law decay for *ω*_*b*_ < *ω* < *ω*_*γ*_ before the exponential falloff above *ω*_*γ*_. Since *ω*_*b*_ approximately scales linearly with *a*_0_ rather than with the ${a}_{0}^{3}$ dependence of *ω*_*γ*_, the increase in the range of high-order harmonics that can be efficiently generated is not as rapid as the naive CSE model predicts. Nonetheless, for experimentally reachable conditions, efficient keV photon production is possible.

Although previous work has limited CSE models to thin foils in reflection^46^ or transmission^30,47,62^, or highly-oblique p-polarized incidence^45^, we have found that when the bunch width is treated appropriately, the CSE model is valid across a wide range of conditions and that both *ω*_*b*_ and *ω*_*γ*_ are important for many reachable parameters. In short, we have generalized the CSE model to explain the evolution of the reflected harmonic spectrum observed in a large number of particle-in-cell simulations spanning a comprehensive set of interaction parameters, developing scaling relationships between the interaction parameters and the efficiency of harmonic and attosecond pulse generation. The scaling laws developed here point to relativistic high-order harmonic generation driven by petawatt-class lasers as the brightest extreme-ultraviolet and soft x-ray source, and competitive with free-electron lasers for peak power at few-keV photon energies.

## Methods

Using the PIC codes EPOCH^63^ and BOPS^64^, we examined the interaction of single-cycle pulses with fully-ionized overdense (*N* > 1) semi-infinite plasma at relativistic intensities (*a*_0_ > 1) to find *p* and *ω*_*c*_. To determine the underlying scaling behavior, target densities and laser intensities were allowed to vary somewhat outside of realistic parameters. Here we neglect quantum electrodynamic (QED) effects, even for light fields with very large nominal *a*_0_. Although the neglect of QED for large *a*_0_ is not physical, it allows us to determine the "relativistic limit” of a particular set of conditions; if we fix *a*_0_/*N*, electron trajectories approach an asymptote as *a*_0_ → *∞*. This limit provides an idealized case which more rigorously validates the underlying mechanism. At more realistic conditions, *a*_0_ ≈ 1 effects can obscure the relativistic mechanism and simulations results may not unambiguously distinguish models, even when those models make different predictions. With a mechanism validated by the relativistic limit, we can treat more realistic values of *a*_0_ – where there are no QED effects – as small deviations from the ideal relativistic case. The key physics of relativistic RHHG can be captured with a one-dimensional treatment; for non-zero laser angle of incidence (*θ*_*L*_) a relativistic transform to a boosted reference frame (Bourdier method^65^) removed dependence on the second spatial dimension. Although the spatial axis is one-dimensional, all three components of the velocity are calculated, and two-dimensional trajectories are found by integration of the transverse velocity components. The simulations presented here used spatial resolutions between 3,000 and 50,000 cells per laser wavelength, with at least 10 cells/wavelength for the shortest harmonic wavelength from which any conclusions were drawn. The number of particles/cell was varied based on noise requirements and ensuring that the critical density was resolved; up to 5000 particles/cell were used. The reflected spectra were found by taking the Fourier transform of the reflected fields. Filtered attosecond pulses were found in a spectral range *ω*_*LF*_ < *ω* < *ω*_*UF*_ from the inverse Fourier transform. In all cases, the pulse envelopes were taken as Gaussian.

### Calculation of power law exponent

The exponent *p* of the spectral power law [*I*(*ω*) ∝ *ω*^−*p*^] is found by a linear fit of the PIC-calculated spectrum. The slope of the logarithm of the intensity vs the logarithm of the frequency (${{\rm{\log }}}_{10}I=-\,p{{\rm{\log }}}_{10}\omega +C$) is the exponent associated with the power-law. The coefficient of proportionality becomes the additive constant *C*. The fit is based on a particular interval of the spectrum: e.g. in Fig. 4  *p* is calculated based on the range 1 < *ω*/*ω*_*L*_ < 100. Since the entire spectrum – not just the harmonic peaks – is used, spectra with distinct harmonic structure will be fit by a line that passes below their harmonic maxima. Note that the range of the fit also means that the fundamental frequency does not dominate or constrain the fit, and in some cases there may be a noticeable discrepancy between the power-law fits and the calculated spectra at *ω*/*ω*_*L*_ ≈ 1.
